# Underscreened Women Remain Overrepresented in the Pool of Cervical Cancer Cases in Spain: A Need to Rethink the Screening Interventions

**DOI:** 10.1155/2015/605375

**Published:** 2015-06-09

**Authors:** Raquel Ibáñez, María Alejo, Neus Combalia, Xavier Tarroch, Josefina Autonell, Laia Codina, Montserrat Culubret, Francesc Xavier Bosch, Silvia de Sanjosé

**Affiliations:** ^1^Unit of Infections and Cancer, Cancer Epidemiology Research Programme, IDIBELL, Catalan Institute of Oncology (ICO), Avenida Gran Vía 199-203, L'Hospitalet de Llobregat, 08908 Barcelona, Spain; ^2^Pathology Department, Hospital General de L'Hospitalet, L'Hospitalet de Llobregat, 08906 Barcelona, Spain; ^3^Pathology Department, Corporació Sanitaria Parc Tauli, Sabadell, 08208 Barcelona, Spain; ^4^Pathology Department, Hospital Mutua de Terrassa, Terrassa, 08221 Barcelona, Spain; ^5^Pathology Department, Consorci Hospitalari de Vic, Vic, 08500 Barcelona, Spain; ^6^Pathology Department, Hospital d'Althaia, Manresa, 08243 Barcelona, Spain; ^7^Pathology Department, Consorci Sanitari de Terrassa, Terrassa, 08227 Barcelona, Spain; ^8^CIBER Epidemiology and Public Health, 08036 Barcelona, Spain

## Abstract

*Objective*. Audit of women with invasive cervical cancer (CC) is critical for quality control within screening activities. We analysed the screening history in the 10 years preceding the study entry in women with and without CC during 2000–2011. *Methods*. 323 women with CC from six pathology departments in Catalonia (Spain) and 23,782 women with negative cytology were compared. Age, previous history of cytologies, and histological type and FIGO stage were collected from the pathology registries. Logistic regression analysis was used to estimate odds ratios (OR) and 95% confidence intervals (CI95%). *Results*. History of cytology was registered in 26.2% of CC cases and in 78% of the control women (*P* < 0.0001) and its frequency decreased with increasing age. Compared to women with squamous cell carcinoma, adenocarcinoma cases were significantly more likely to have a cytology within the 3-year interval preceding cancer diagnosis (OR = 2.6 CI 95%: 1.2–5.6) and to have normal cytology results in previous screenings (OR = 2.4 CI 95%: 1.2–4.5). FIGO II–IV cases were more common among older women (older than 60 years). *Conclusions*. Absence of prior screening history was extremely common among CC cases compared to controls. Organized actions to reduce underscreened women and use of highly sensitive HPV-based tests could be important to reduce CC burden.

## 1. Introduction

The major cause of cervical cancer (CC) is the persistent infection with oncogenic types of human papillomavirus (HPV) [1]. CC is preceded by visible morphological cervical intraepithelial lesions (CIN) that can be detected through regular exam of exfoliated cells of the cervix. Although vaccines to prevent infection with specific oncogenic HPVs are now available, it will take at least 2-3 decades for their effects on CC burden to be seen [2].

Meanwhile, adult unvaccinated women remain target for CC screening. However, to guarantee an adequate population impact of screening, large population coverage and adequate follow-up have been fundamental in decreasing incidence and mortality of CC [3, 4].

In the autonomic region of Catalonia, CC screening is opportunistic. Efforts to increase CC screening coverage were initiated in 2006 within the public sector with the introduction of new screening protocol [5]. Routine screening with cervical cytology is recommended in the region to women aged 25–65 with a 3-year interval. Cervical cytology coverage was estimated for the period 2008–2011 to be around 70% if public as well as private coverage is considered [6, 7]. Every year, there will be around 378 new cancer cases of CC in the region implying a lifetime risk of one out of 106 women [8].

As part of quality assessment of the screening activities in the region, we aimed to monitor screening uptake among women who have developed invasive CC. For this purpose, we analysed the screening history in the 10 years preceding the study entry among women with and without CC who attended within the Public Health System from a predefined study area for the period 2000–2011.

## 2. Patients and Methods

### 2.1. Data Collection

The study includes all women with an incident invasive CC diagnosed in six pathology departments of Catalonia during January 2000 to December 2011 (Hospital General de L'Hospitalet, Corporació Sanitaria Parc Taulí, Hospital Mutua de Terrassa, Consorci Hospitalari de Vic, Hospital Althaia, and Consorci Hospitalari de Terrassa). The aforementioned hospitals encompass 2 of the 7 health counties that compose Catalonia's Health System. The female population over the age of 24 in the area was 306,008 women [9].

A total of 323 newly diagnosed CC cases were identified during the study period. Information collected from clinical records during the 10 years prior to CC diagnosis included history of previous cytologies, time since the last cytology to cancer diagnosis, age of the patient at time of cancer diagnosis, and type and stage of CC. We assumed that cytologies taken within the previous 6 months to the cancer diagnosis were obtained as part of the diagnostic process and excluded them for the analysis. Women were categorised as never screened if there was no record on cervical cytology.

A comparison group consisted of 23,782 women with a normal cytology retrieved from one of the six pathology departments and resident in the study area in 2007. Thereafter, these women are referred to as control group. Information about the presence or absence of prior cytologies during 10 years before negative cytology and ages of the women were collected from the same source as the cases.

The overall project was approved by the ethical committee of the Catalan Institute of Oncology. Any information regarding the identification of patients was anonymized before analysis.

### 2.2. Screening Tests and Stage of Cervical Cancers

In all centres, conventional cytology was used. All the cytological results were classified or adapted if needed, according to the 2001 Bethesda system [10].

CC cases were staged according to the International Federation of Gynaecology and Obstetrics (FIGO) classification system [11].

### 2.3. Statistical Analysis

For the women with CC, information consisted of histological type, FIGO stage (unknown, I, II, III, and IV), and registration to previous screening, which included result of prior cytology (no previous cytology, normal or abnormal), time since the last previous cytology (≤3 years and >3 years before cancer diagnostic according to established 3-year screening interval), and numbers of previous cytologies (without, 1, or >1 prior cytology). The large majority of cases were squamous cell carcinoma (SCC) or adenocarcinoma (ADC). The remaining cases (*N* = 15) were reclassified as follows: three clear cell adenocarcinoma and one adenoidcystic carcinoma cases were included in the ADC group while one small cell carcinoma and ten adenosquamous carcinoma were included in the SCC group. Data were analysed with and without these rare histological types and the result was similar in both situations, so they were included in the analysis. Women with nonevaluable previous cytology (*N* = 2) or missing age were excluded (*N* = 2).

Logistic regression was performed to estimate the odds ratio (OR) with the corresponding 95% confidential intervals (CI 95%) of developing ADC or SCC. Adjustment was done by geographical area and women's age.

Differences in the presence of cervical cytology in the previous ten years between the CC cases and the control group were estimated taking into account the different age structure of both groups. Proportional differences were compared using chi-square test. Statistical significance was defined as *P* < 0.05.

## 3. Results

Table 1 shows the age distribution and the percentage of women with prior cytology during the previous 10 years among 323 CC cases and 23,782 control women. Women with CC were on average 12.6 years older than control women (54.4 versus 41.9). History of previous cytology was identified in 78.8% of the control women and in 26.2% of CC cases (*P* < 0.0001). After adjustment for differences in the age structure, the global use of prior cytology among CC was 70% lower compared to that in controls.

Table 2 describes the age distribution, the period, and the FIGO stages at diagnosis of the 323 CC cases by histological type. Overall, 248 (76.8%) of the CC were SCC and 75 (23.2%) were ADC. The average age was 54.4 years with a range of 23–96 years. The majority of the CC were diagnosed in the age range of 40 to 49 years (26.2%). In 40.9% of CC cases, the cancer stage was unknown. No statistically significant differences were observed between histological types and the general characteristics explored.

Women with ADC were significantly more likely to have had a prior cytology (OR = 2.1 CI 95%: 1.2–3.8), more than 1 previous cytology (OR = 3.2 CI 95%: 1.5–6.5), a cytology 3 years before cancer diagnosis (OR = 2.6 CI 95%: 1.2–5.6), and a normal cytology (OR = 2.4 CI 95%: 1.2–4.5) as compared to women with SCC (Table 3).

**Figure 1:**
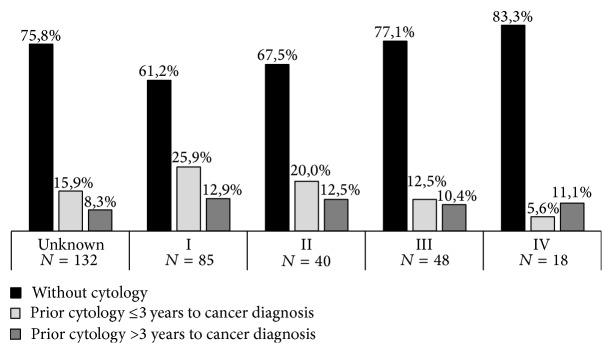
Distribution of FIGO stages by time since prior cytology.

Age was strongly associated with FIGO stages (*P* < 0.05) (Table 4). Women aged less than 40 years were more likely to have a stage I CC while stages II–IV were more common among women aged 60 or more. Older women were less likely to have a prior cytology (82.9% and 79.2% in age groups of 60–69 and ≥70 years, resp.) or to have had a cervical cytology within an interval longer than 3 years. In the presence of a previous screening history, women younger than 40 years old were more likely to have an abnormal cytological result compared to older women (*P* = 0.05). Women with normal cytology were, on average, older than women with an abnormal cytology (54.6 versus 43.8 years, resp., *P* = 0.003). Most of the atypical squamous cell of undetermined significance (ASC-US), atypical squamous cells cannot exclude a high grade squamous intraepithelial lesion (ASC-H) and atypical glandular cells of undetermined significance (AGC) results were diagnosed in the group of women aged 40–49 years (33.3%) while the low grade squamous intraepithelial lesions (LSIL) results were mostly diagnosed in women younger than 40 years (85.7%). About half of the negative cytologies (56.7%) and 80% of the positive cytologies were performed within 3 years prior to CC diagnosis (*P* = 0.029) (data not shown). Among all cases, 12 were in women younger than 30 years of age. Of them, 66.7% did not have any previous cytology, nearly half of the cases (41.6%) were diagnosed in stage I, and four were ADC as histological type.  Figure 1 shows the distribution of the time since previous cytology to cancer diagnosis by FIGO stages. Although the differences were not statistically significant, women without previous cytology were more likely to be diagnosed at more advanced stages.

## 4. Discussion

Our study confirms that lack of CC screening history was substantially common among women with CC. While 73.8% of women with CC did not have a previous cervical cytology record within the Public Health System, less than a quarter of women attending screening with a normal cytological result had no previous cytology data in the previous ten years. The natural history of invasive CC, a disease with long preneoplastic changes, more than 10 years in the majority of the cases, generally allows its early detection. Our comparison with the women attending CC screening having a negative cytology shows clearly a different behaviour towards screening. Although our study was not designed to be a case-control study, it shows, in our opinion, that lack of screening is an outstanding feature among women with CC.

The proportion of unscreened women among CC cases in our study was significantly higher than the 23%–68% range reported by others [12–24]. Organized screening programs consistently have lower percentages when compared to opportunistic screening situations [25–27]. van der Aa et al. [26] from the Netherlands reported a twofold increased risk of death comparing women with CC screened through an opportunistic approach with those detected by organized screening programs. In most studies, advanced age was an additional factor that contributed to absence of screening [16, 17, 19, 21, 22]. In our study, women with no prior history of cytology were significantly older than women with a history of screening (55.8 versus 51.1 years) and over a third of CC cases were diagnosed in women aged over 60. It was in this age group that over 80% of the women had no previous screening history. A recent study about screening coverage in Catalonia [7] confirmed the poor screening history of women aged between 66 and 69 years with only 16% of them reporting a prior cytology in the 3 years prior to the evaluation [7].

Advanced age has consistently been associated with increased disease stages in consistence with our observations where the cases in stages III-IV were double among women aged over 65 years compared to women younger than 40 years old. Most likely, the absence of an adequate screening history plays a major impact in these observations although we cannot rule out that a certain proportion of cases could be newly developed after the end of the screening recommendations [15, 21, 26]. Efforts to reduce this group of underscreened women have been recommended [28]. We have now an ongoing program to actively identify this population [5]. A recent evaluation of this strategy [29] showed that underscreened women had a high burden of cervical disease. Attempts to extend these initiatives into an organized activity are undergoing [30] a randomized trial inviting these women to participate in the screening program.

In contrast with the above data, the utility of CC screening in women younger than 30 years old is questionable given the probability of regression of precancer lesions and the potential harm of the interventions [31]. In our study, 3.7% (*n* = 12) of the women were younger than 30 years of age. According to population-based cancer registries, the specific rate of CC in women aged 20–24 and 25–29 years for the year 2007 was 2.37/100,000 and 5.09/100,000 women, respectively, with a total specific rate of 1.70/100,000 among those younger than 30 years old [8]. One study carried out in Canada [31] among women aged 15 to 29 concluded that CC in adolescents (15–19 years old) was rare and does not justify a population-based screening. Castañón et al. [32] reported, in a study carried out in 1,800 women diagnosed with CC at ages 20–29 from England, that most cases were detected with microinvasive cancer (stage 1A) with excellent prognosis and although cancers diagnosed between 20 and 24 years were more likely to be diagnosed at more advanced stages, their frequency was rarer (2% of all the cancers diagnosed in England in 2010) as the majority of the cancers fell between the range of 26–29 years (63.2%). Prophylactic vaccination will likely play an important role in these age groups as it is expected to reduce the incidence of CC substantially [33].

In the present study, among women with prior cytology, 37% had a previous normal cytology in the 3-year screening interval prior to CC diagnosis. In Andrae et al. [15], 24% of all cases had developed CC despite having a normal cytology within the recommended interval but the percentage increased to 40% for women aged older than 65 years. We could only review a small fraction of previous cytologies, but, in a second reading, three out of 30 could be considered to be false negatives, two of them being among cases with a diagnosis of ADC group and one being with a SCC, confirming the poor sensitivity of a single cervical cytology [22, 25, 34, 35]. The use of HPV testing is now being proposed in many settings for its better prediction of CIN2+ cases [22, 35, 36].

It is well recognized that women with ADC have higher risk of having a previous negative cytology [25, 26, 37–39]. In our study, women with ADC were twice more likely to have a previous negative cytology than women with a SCC. Besides, of all ADC, 17.8% had a prior cytology within a period not exceeding 3 years before cancer diagnosis while this proportion was 9.9% in SCC. Glandular lesions can be missed, especially when they do not involve the transformation zone but are located higher in the endocervical canal. Despite the wide use of cervical brushes that have improved the capture of endocervical cells, the risk of underdetection is likely to remain. HPV testing as primary screening seems to be highly recommended to improve overall sensitivity of screening and in particular for the optimization of adenocarcinoma diagnosis [20].

In a much lower proportion, CC cases could be attributable to poor follow-up [25]. In our study, 40% of the cancers with previous cytology had a result of LSIL/HSIL and most of them (80% of LSIL and 60% of HSIL) were diagnosed in a period of 3 years or lower at cancer diagnosis. The reasons why these women did not have an adequate follow-up are unknown. In a certain proportion, women refrained from follow-up suggesting that the adequacy of the message is probably not optimal [40]. Others have reported that a potential cause of loss of follow-up is a repeated negative cytology or negative colposcopy [15, 35], suggesting that a single negative test at follow-up is not enough to send back women to regular screening when there is a positive test.

The proportion of ADC and SCC found in our study did not differ from other studies [12–24]. Cytological results of ASC-US, AGC and ASC-H were found in 4.4% of SCC while this percentage was more frequent in ADC cases (9.3%). Despite small numbers (3/75), AGC was only seen in the ADC group and all of them had been diagnosed within the 3-year screening interval. Cytologies classified as AGC, although relatively uncommon, are likely to be a reliable marker of cancer varying the incidence of cases from 0.05% to 2.1 [41] suggesting that immediate colposcopy referral is probably the best option for these women [39, 41].

There is much controversy about what is the appropriate age of stopping screening. A case-control study carried out by Castañón et al. [42] suggested that women with adequate negative screening at the age of 50–64 years substantially reduced their risk of CC at the age of 65 years and older compared with women who were not screened. However, the magnitude of that protection decreased with time since the last screen, recommending exiting the screening only if the last three tests were all negatives. In our data, 81% of women aged 65 years or more did not have any previous cytology registered. Among the 18 women with a previous cytology, 13 had a negative cytology performed over 3 years of CC diagnosis and only 4 women had a negative cytology preceding CC diagnosis within the 3-year screening interval. Interestingly, 3 of these 4 women were diagnosed with ADC, in agreement with the poorer sensitivity of cytology in the diagnosis of glandular lesions as compared to squamous ones. Our data suggest that the number of CC cases that occurred in women over the age of 65 when exiting the screening following the recommendations is likely to be very small.

### 4.1. Study Limitations and Strengths

We have been able to explore screening history among women attending the Public Health System. It is unknown if women attended within the private gynaecology sector have a different behaviour and, thus, we cannot extrapolate our results to them. Our control group consisted of a large sample of women without CC diagnosis and normal cytology for the purpose to contrast the absence of screening history by age group in women with and without CC. We cannot exclude that additional explanations due to factors other than age could partially explain the huge difference in screening uptake.

Unfortunately, our data was limited in relation to poor knowledge on stage of disease. This is explained by the fact that we used pathology registries and not clinical records where stage is likely to be more complete.

We could only review the negative previous cytologies for the period 2000–2007 due to logistic limitations but no major changes have taken place in cervical cytology guidelines in the region for the recent years. Thus, we think that the percent of false negative results should be similar across the years evaluated.

Strengths of this study are that information was reported by six hospitals covering a predeterminate geographical area. No differences in the data collected were observed between them. The control population, despite not being matched to the cases by year of diagnosis, was composed of 23,782 women, providing a robust indication of the screening uptake when age adjusted analysis is presented.

## 5. Conclusions

In summary, the results of this study indicate that lack of screening is a major limitation in CC prevention. Efforts to increase population coverage of screening, especially in older women, in which a high number of nonscreened and higher stages of cancer were observed, have to be paired with improving the sensitivity of the principal screening test for a better CC diagnosis. Use of HPV-based screening tests may significantly improve the efficiency of screening interventions.

## Figures and Tables

**Table 1:** Age and history of previous screening cytology in women with cervical cancer and women with a normal cytology.

	Control women		Women with cervical cancer^*∗*^		*P* value
	*N* = 23,782	% column	*N* = 313	% column	
Age					
<30 years	5.224	22,0%	12	3,9%	<0,0001
30–39 years	5.879	24,7%	56	18,0%	
40–49 years	5.426	22,8%	84	27,0%	
50–59 years	4.277	18,0%	47	15,1%	
60–69 years	2.378	10,0%	41	13,2%	
≥70 years	598	2,5%	71	22,8%	
With at least one cytology in the last 10 years^*∗∗*^					
With cytology	18.733	78,8%	82	26,2%	<0,0001
Without cytology	5.049	21,2%	231	73,8%	

Age-standardized coverage ratio					0,3

^*∗*^There are 2 ages missing.

^*∗∗*^10 women with cervical cancer had a prior cytology performed over 10 years ago and they were excluded for the analysis.

**Table 2:** General characteristics of the study population by histological types.

General characteristics	Total *N* = 323	%	Histological type				
			Squamous carcinoma		Adenocarcinoma		*P* value
			*N* = 248	% column	*N* = 75	% column	
Age^*∗*^							
<30 years	12	3,7%	8	3,3%	4	5,3%	0,62
30–39 years	56	17,4%	43	17,5%	13	17,3%	0,54
40–49 years	84	26,2%	65	26,4%	19	25,3%	0,54
50–59 years	51	15,9%	43	17,5%	8	10,7%	0,11
60–69 years	41	12,8%	31	12,6%	10	13,3%	0,75
≥70 years	77	24,0%	56	22,8%	21	28,0%	0,64
Year of diagnosis							
2000–2003	119	36,8%	92	37,1%	27	36,0%	0,44
2004–2007	73	22,6%	60	24,2%	13	17,3%	0,17
2008–2011	131	40,6%	96	38,7%	35	46,7%	0,37
FIGO stages							
Unknown stage	132	40,9%	101	40,7%	31	41,3%	0,70
I	85	26,3%	63	25,4%	22	29,3%	0,78
II	40	12,4%	31	12,5%	9	12,0%	0,49
III	48	14,9%	37	14,9%	11	14,7%	0,56
IV	18	5,6%	16	6,5%	2	2,7%	0,17

^*∗*^There are 2 ages missing.

All the variables are adjusted by area and groups of age.

FIGO: International Federation of Gynecology and Obstetrics.

**Table 3:** Screening history of the study population by histological type.

Screening history	Total *N* = 323	%	Histological type							
			Squamous carcinoma		Adenocarcinoma		*P* value	OR	CI 95%	
			*N* = 248	% column	*N* = 75	% column				
Previous cytologies to cancer diagnosis^*∗*^										
Without prior cytology	231	71,5%	187	75,4%	44	58,4%	0,01	Reference		
With prior cytology	92	28,5%	61	24,6%	31	41,3%		2,1	1,2	3,8
Number of previous cytologies^*∗*^										
Without prior cytology	231	71,5%	187	75,4%	44	58,7%	0,01	Reference		
1 previous cytology	50	15,5%	37	14,9%	13	17,3%	0,31	1,5	0,7	3,1
>1 previous cytologies	42	13,0%	24	9,7%	18	24,0%	0,00	3,2	1,5	6,5
Time between previous cytology and cancer diagnosis^*∗*^										
Without prior cytology	231	71,5%	187	75,4%	44	58,7%	0,03	Reference		
Prior cytology ≤3 years before cancer diagnostic	58	18,0%	40	16,1%	18	24,0%	0,07	1,9	0,9	3,7
Prior cytology >3 years before cancer diagnostic	34	10,5%	21	8,5%	13	17,3%	0,02	2,6	1,2	5,6
General results of previous cytologies^*∗*^										
Without prior cytology	231	72,0%	187	76,0%	44	58,7%	0,02	Reference		
Normal	60	18,7%	38	15,4%	22	29,3%	0,01	2,4	1,2	4,5
Abnormal	30	9,3%	21	8,5%	9	12,0%	0,18	1,8	0,8	4,4
ASC-US-AGC-H^a^	18	60,0%	11	52,4%	7	77,8%				
LSIL^a^	7	23,3%	6	28,6%	1	11,1%				
HSIL^a^	5	16,7%	4	19,0%	1	11,1%				

^*∗*^Cytologies taken within the previous 6 months to the cancer diagnosis were excluded for the analysis because they were considered as part of the diagnostic process. There are 2 women with a nonevaluable previous cytology. These cases were excluded for the analysis in which result of previous cytology is involved.

^a^Percentages of specific cytological abnormalities are among the abnormal cytologies.

All the variables are adjusted by area and groups of age.

ASC-US: atypical squamous cell of undetermined significance, ASC-H: atypical squamous cells which cannot exclude a high grade squamous intraepithelial lesion, AGC: atypical glandular cells of undetermined significance, LSIL: low grade squamous intraepithelial lesion, and HSIL: high grade squamous intraepithelial lesion.

**Table 4:** Screening history of the study population by groups of age.

Screening history	Age^*∗*^												
	<30 years		30–39 years		40–49 years		50–59 years		60–69 years		>70 years		*P* value *x* ^2^
	*N* = 12	% column	*N* = 56	% column	*N* = 84	% column	*N* = 51	% column	*N* = 41	% column	*N* = 77	% column	
FIGO stage													
Unknown	4	33,3%	20	35,7%	39	46,4%	19	37,3%	19	46,3%	30	39,0%	0,02
I	7	58,3%	24	42,9%	27	32,1%	10	19,6%	7	17,1%	10	13,0%	
II	0	0,0%	2	3,6%	7	8,3%	11	21,6%	4	9,8%	16	20,8%	
III	1	8,3%	7	12,5%	9	10,7%	6	11,8%	8	19,5%	16	20,8%	
IV	0	0,0%	3	5,4%	2	2,4%	5	9,8%	3	7,3%	5	6,5%	
Cancer diagnosis													
Squamous carcinoma	8	66,7%	43	76,8%	65	77,4%	43	84,3%	31	75,6%	56	72,7%	0,69
Adenocarcinoma	4	33,3%	13	23,2%	19	22,6%	8	15,7%	10	24,4%	21	27,3%	
Previous cytologies to cancer diagnosis													
Without prior cytology	8	66,7%	36	64,3%	59	70,2%	31	60,8%	34	82,9%	61	79,2%	0,10
With prior cytology	4	33,3%	20	35,7%	25	29,8%	20	39,2%	7	17,1%	16	20,8%	
Within those with previous cytology^*∗∗*^													
Number of previous cytologies													
1 previous cytology	1	25,0%	10	50,0%	14	56,0%	12	66,7%	2	28,6%	9	56,3%	0,48
>1 previous cytologies	3	75,0%	10	50,0%	11	44,0%	6	33,3%	5	71,4%	7	43,8%	
Time between previous cytology and cancer diagnosis													
Prior cytology ≤3 years before cancer diagnostic	3	75,0%	17	85,0%	18	72,0%	12	66,7%	4	57,1%	4	25,0%	0,01
Prior cytology >3 years before cancer diagnostic	1	25,0%	3	15,0%	7	28,0%	6	33,3%	3	42,9%	12	75,0%	
Results of previous cytologies													
Normal	1	25,0%	10	50,0%	17	68,0%	12	66,7%	5	71,4%	15	93,8%	0,05
Abnormal	3	75,0%	10	50,0%	8	32,0%	6	33,3%	2	28,6%	1	6,3%	
ASC-US-AGC-H^a^	1	33,3%	4	40,0%	6	75,0%	4	66,7%	2	100,0%	1	100,0%	
LSIL^a^	2	66,7%	4	40,0%	0	0,0%	1	16,7%	0	0,0%	0	0,0%	
HSIL^a^	0	0,0%	2	20,0%	2	25,0%	1	16,7%	0	0,0%	0	0,0%	

^*∗*^There are 2 ages missing.

^*∗∗*^Cytologies taken within the previous 6 months to the cancer diagnosis were excluded for the analysis because they were considered as part of the diagnostic process. There are 2 women with a nonevaluable previous cytology. These cases were excluded for the analysis in which result of previous cytology is involved.

^a^Percentages of specific cytological abnormalities are among the abnormal cytologies.

FIGO: International Federation of Gynecology and Obstetrics, ASC-US: atypical squamous cell of undetermined significance, ASC-H: atypical squamous cells cannot exclude a high grade squamous intraepithelial lesion, AGC: atypical glandular cells of undetermined significance, LSIL: low grade squamous intraepithelial lesion, and HSIL: high grade squamous intraepithelial lesion.
